# 3D-Printed Tumor Phantoms for Assessment of *In Vivo* Fluorescence Imaging Analysis Methods

**DOI:** 10.1007/s11307-022-01783-5

**Published:** 2022-10-28

**Authors:** Ethan P. M. LaRochelle, Samuel S. Streeter, Eammon A. Littler, Alberto J. Ruiz

**Affiliations:** 1 QUEL Imaging, 85 N. Main Street Suite 142, White River Junction, VT 05001 USA; 2 Thayer School of Engineering at Dartmouth, 14 Engineering Drive, Hanover, NH 03755 USA

**Keywords:** Fluorescence guided surgery, Optical phantom, Surgical navigation, Standards, ICG

## Abstract

**Purpose:**

Interventional fluorescence imaging is increasingly being utilized to quantify cancer biomarkers in both clinical and preclinical models, yet absolute quantification is complicated by many factors. The use of optical phantoms has been suggested by multiple professional organizations for quantitative performance assessment of fluorescence guidance imaging systems. This concept can be further extended to provide standardized tools to compare and assess image analysis metrics.

**Procedures:**

3D-printed fluorescence phantoms based on solid tumor models were developed with representative bio-mimicking optical properties. Phantoms were produced with discrete tumors embedded with an NIR fluorophore of fixed concentration and either zero or 3% non-specific fluorophore in the surrounding material. These phantoms were first imaged by two fluorescence imaging systems using two methods of image segmentation, and four assessment metrics were calculated to demonstrate variability in the quantitative assessment of system performance. The same analysis techniques were then applied to one tumor model with decreasing tumor fluorophore concentrations.

**Results:**

These anatomical phantom models demonstrate the ability to use 3D printing to manufacture anthropomorphic shapes with a wide range of reduced scattering (*μ*_*s*_′: 0.24–1.06 mm^−1^) and absorption (*μ*_*a*_: 0.005–0.14 mm^−1^) properties. The phantom imaging and analysis highlight variability in the measured sensitivity metrics associated with tumor visualization.

**Conclusions:**

3D printing techniques provide a platform for demonstrating complex biological models that introduce real-world complexities for quantifying fluorescence image data. Controlled iterative development of these phantom designs can be used as a tool to advance the field and provide context for consensus-building beyond performance assessment of fluorescence imaging platforms, and extend support for standardizing how quantitative metrics are extracted from imaging data and reported in literature.

**Supplementary Information:**

The online version contains supplementary material available at 10.1007/s11307-022-01783-5.

## Introduction

Interventional fluorescence imaging is increasingly being utilized to quantify cancer biomarkers in both clinical and preclinical models, yet absolute quantification is complicated by many factors. The field of fluorescence-guided surgery (FGS) has predominantly focused on perfusion assessment using indocyanine green (ICG) [1]; however, other targeted contrast agents have recently been approved by the FDA [2–4]. While surgeons can easily use ICG to visualize perfusion, targeted fluorophores introduce new complexities for the purpose of assessing surgical margins [5]. Unlike PET imaging, which can directly relate detected signal to the administered dose, quantitative fluorescence imaging must account for (1) invariable system parameters, (2) tissue properties, (3) operator variabilities, and (4) attributes specific to the contrast probe [6]. Even with these confounding factors, semi-quantitative fluorescence imaging is currently used in both preclinical and clinical studies [7–10]. While these studies may be repeatable within an institution, multi-center reproducibility and broader adoption of quantitative fluorescence imaging requires additional consensus within the field. Beyond converging on system characterization methodologies using standardized reference targets, anthropomorphic phantoms can be used to build consensus on how to implement and report image analysis metrics.

Efforts to standardize fluorescence imaging have primarily been an academic discussion and are gaining support through involvement with professional organizations such as American Association of Physicists in Medicine (AAPM) Task Group 311 (TG311) [11, 12], European Society for Molecular Imaging (ESMI) Study Group on Imaging Standardization, and World Molecular Imaging Society (WMIS) Optical Surgical Navigation (OSN) Interest Group. These groups are working to identify methods for system characterization and comparison to enable multi-center studies. A common recommendation is the use of reference targets and phantoms to advance these goals. While the primary purpose of these targets is for system performance assessment, similar techniques can be used to help develop consensus around reporting metrics extracted from *in vivo* fluorescence images.

A recent review identified eight properties required for a biophotonic phantom: (1) tissue-like properties, (2) tunability, (3) stability, (4) architectural flexibility, (5) reproducibility, (6) simple maintenance, (7) safety, and (8) availability [13]. Serial dilutions of fluorescent contrast agents can be prepared with tissue optical properties and used for system characterization; however, this method is prone to inter-user reproducibility errors, short shelf-life, and may require a dedicated preparation space to satisfy environmental hazard requirements. Solid composite phantoms provide the opportunity for extended shelf stability and multi-center distribution [14, 15]. A solid standardization reference phantom was proposed in 2016 and has been subsequently revised in recent years [6, 16, 17]. This phantom utilizes quantum dots embedded in tissue-simulating polyurethane wells to quantify system performance metrics needed to enable inter-laboratory comparisons. This concept was extended in 2020 with a modular design to address manufacturability and utilized an ICG-equivalent fluorophore in place of quantum dots [14]. To further address design flexibility, a resin-based 3D printing technique was developed [15]. While the primary focus of many of the solid composite reference phantoms have been to provide engineers with detailed system characterization, 3D printing provides the ability to manufacture more complex shapes with controlled, homogeneous optical properties. The development of anthropomorphic phantoms with tunable optical properties can address assessment challenges observed with *in vivo* and *ex vivo* quantitative fluorescence imaging.

Here, we present three anthropomorphic phantoms designed for the performance assessment of tumor visualization sensitivity for fluorescence guidance imaging systems. The target anatomy was chosen primarily to demonstrate wide tunability of tissue optical properties for both embedded and surface tumors. Each phantom incorporates near-infrared (NIR) fluorescent tumor tissue with discrete boundaries embedded within material of representative tissue optical properties. Phantoms with fixed tumor fluorophore concentration were first imaged by two fluorescence imaging systems using two methods of image segmentation and four assessment metrics to demonstrate variability in the quantitative assessment. Additional tumor fluorophore concentrations for one model were imaged on a third system to demonstrate trends in analysis metrics as a function of fluorophore concentration. The results demonstrate the variability extending beyond system performance characterization to highlight how image analysis techniques influence reported sensitivity metrics, highlighting the need for convergence in reporting methodologies.

## Methods

Solid tumor phantoms relevant to three different anatomical sites representative of a range of optical properties were developed. The three tumor models were based on a micro-CT scan of an *ex vivo* breast lumpectomy specimen (ClinicalTrials.gov Identifier: NCT04257799), a CT of a liver tumor [18], and an MRI of a soft-tissue sarcoma [19, 20]. Image data were imported into 3D Slicer (v4.11.20210226) in DICOM format [21]. The Segmentation Editor module was used to prepare each phantom model before exportation as an STL file used for printing [22]. Key printing considerations for the phantoms included making watertight segmentations of the primary tumor and surrounding tissue and slicing the surrounding tissue segmentation in half laterally such that the primary tumor print could be set into the surrounding tissue print. An approximately 0.2-mm margin was eroded from the primary tumor segmentation to ensure an optimal mechanical fit inside the surrounding tissue print. The liver tumor model was reduced by 50% from the original DICOM volume. The tumor dimensions and distance to imaging plane are provided in Table 1. The 3D printer has a nominal resolution of 50 μm, and final feature tolerances were better than 500 μm.

**Table 1:** Dimensions of tumors in anthropomorphic phantom models

Model	Dimensions (mm)	Volume (cm^3^)	Distance to imaging plane (mm)
Breast Tumor	28.5 × 21.0 × 10.9	2.43	2.2
Liver Tumor	15.3 × 14.6 × 15.5	1.34	4.9
Sarcoma	32.8 × 29.5 × 20	7.47	0

The optical properties of the three phantoms (*i.e.*, *μ*_*a*_, *μ*_*s*_′) were determined with reference to a review of optical properties of biological tissues [23]. These optical properties consider the components of multiple chromosomes including blood, lipid, and water over a range of wavelengths, which were tuned at 800 nm due to the emission wavelength of the ICG-based fluorophore. Using stock solutions of absorbing (nigrosin or hemin) and scattering (TiO_2_) particles, we varied the optical properties of the 3D printed materials as described in previous work [15]. Custom resin formulations were developed for each tissue type. Tumor inclusions were printed with embedded 1 μM ICG-equivalent fluorophore [14, 15]. The surrounding bulk material was printed with either no fluorophore or 3% (0.03 μM) ICG-equivalent fluorophore. All 3D printed material was manufactured using a previously published masked stereolithography 3D printing technique [15]. To measure the optical properties, 30 mm × 30 mm × 20 mm blocks with no fluorophore were manufactured using the same resin formulation. A commercial Spatial Frequency Domain Imaging (SFDI) system (Reflect RS, Modulim, Irvine, CA) was used to determine the optical properties of each material. Optical properties were quantified at eight wavelengths (471, 526, 591, 621, 659, 691, 731, and 851 nm) using five spatial frequency patterns (0.00, 0.05, 0.10, 0.15, and 0.20 mm^−1^).

**Fig. 1:**
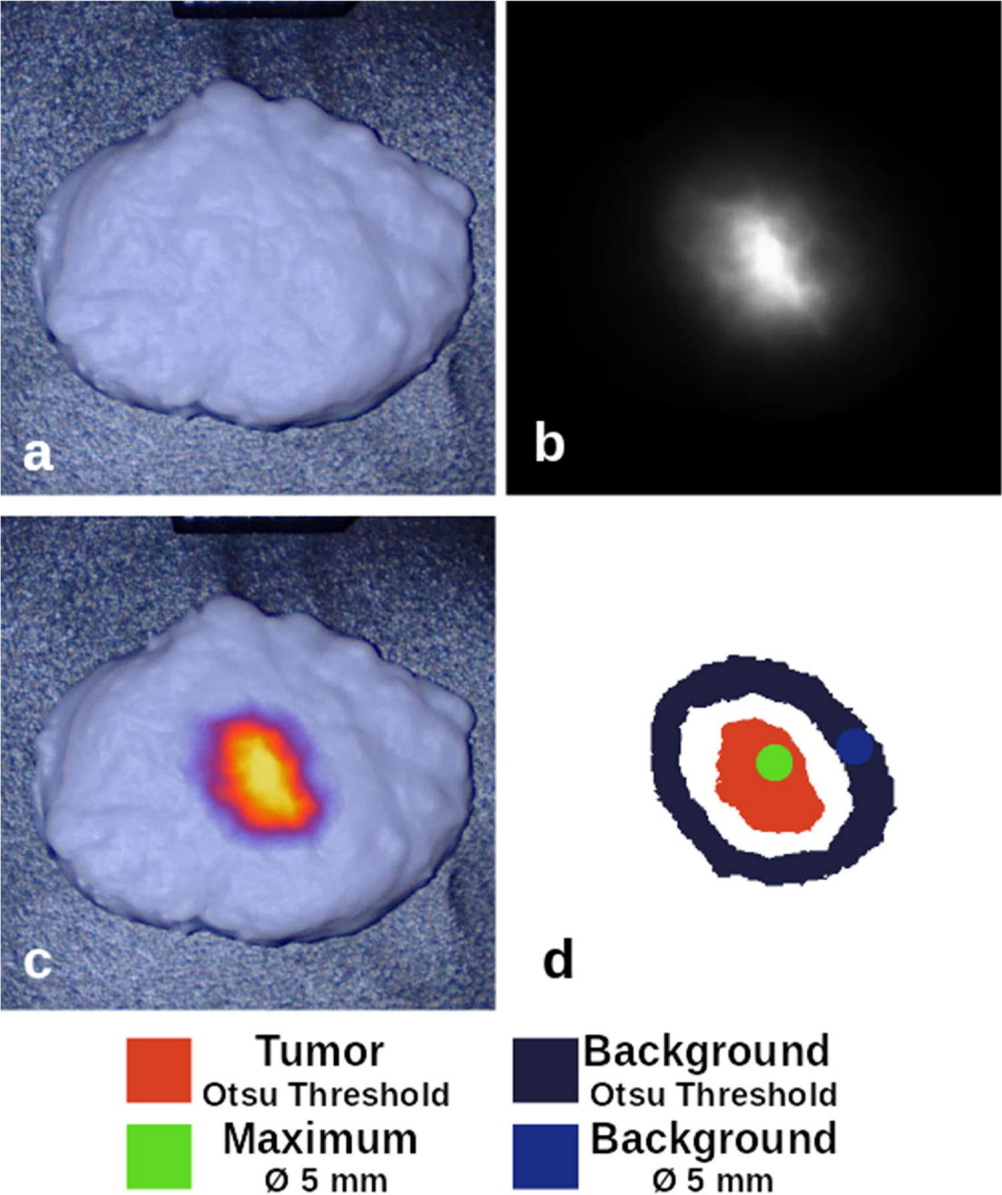
A breast lumpectomy phantom imaged on a clinical fluorescence imaging system in **a** visible white light mode, **b** NIR fluorescence mode, and **c** fused overlay mode. A mask to define regions of interest is shown in **d**, which was used to process the intensity data of the fluorescence image

Fluorescence images were collected using a FLUOBEAM-800 (Fluoptics, Cambridge, MA) and a EleVision IR platform (Medtronic, Minneapolis, MN). The working distance to the bottom surface was 20 cm for the FLUOMEAM and 30 cm for the EleVision. The EleVision NIR-High Contrast mode was used for data collection. The FLUOBEAM uses an excitation wavelength of 750 nm and emission detection with a 800 long pass filter [5], whereas the EleVision uses an 785 nm excitation and 825 nm bandpass filter. The images were analyzed using the Python Scikit Image module [24]. The analysis workflow was semi-automated and cropped each image to a box around each phantom. Since EleVision fluorescence images were saved as a compressed 8-bit RGB image, a grayscale conversion was applied and scaled to between 0 and 1. The FLUOBEAM images were initially 16-bit grayscale images and were converted to the same scale for analysis. Two methods were compared for defining tumor and background regions: first, an Otsu threshold was determined for the cropped image where the tumor was defined as all pixels 150% above this threshold, and the background was defined as all pixels between 10 and 70% of the threshold (Fig. 1). This initial method used these entire regions to determine the mean and standard deviation of pixel intensities. A second method identified a circular 5-mm diameter region centered at the maximum pixel intensity and another region of the same size within the previously defined background region. Four metrics were calculated using the mean and standard deviation identified by each of these methods: (1) signal-to-background ratio (SBR), (2) signal-to-noise ratio (SNR), (3) contrast-to-noise ratio (CNR), and (4) contrast-to-variability ratio (CVR) [25]. The metrics are calculated as SBR = $\frac{{\mu }_{T}}{{\mu }_{B}}$, SNR = $\frac{{\mu }_{T}}{{\sigma }_{B}}$, CNR = $\frac{{\mu }_{T}-{\mu }_{B}}{{\sigma }_{B}}$, and CVR = $\frac{{\mu }_{T}-{\mu }_{B}}{\sqrt{{\sigma }_{T}^{2}+{\sigma }_{B}^{2}}}$, where *µ*_*T*_ and *σ*_*T*_ are the mean and standard deviation of the tumor region of interest (ROI) pixels, and *µ*_*B*_ and *σ*_*B*_ are the mean and standard deviation of the background ROI pixels (Fig. 1).

The same analysis methods were then applied to the breast model containing tumors with decreasing fluoresence concentration (1 μM, 0.3 μM, 0.1 μM, 0.03 μM) of ICG-equivalent fluorophore. Measurements were collected in triplicate after removing and repositioning the phantom in the imaging field. A custom imaging system consisting of a cooled CMOS camera (ASI294, ZWO, China), two beam-expanded 785 nm laser diodes (Stingray, Coherent Inc, California, USA), and an excitation source and 800 nm longpass (Omega Optical, Vermont, USA) emission filter to collect fluoresence images of the phantom configurations. Fluorescence and background image pairs were acquired with a camera gain of 130 and exposure time of 2 s and saved as a16-bit grayscale values. Ambient light was minimized and background images, collected with the laser off, were subtracted from the corresponding fluorescence-mode image. The image analysis used the same Python code where additional ROI sizes (5, 7.5, and 10 mm diameters) were compared.

## Results

**Fig. 2:**
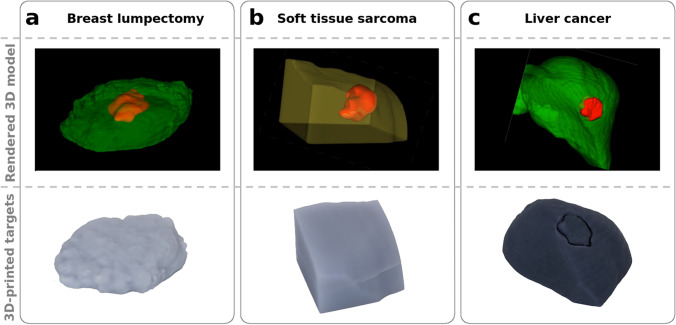
Anthropomorphic models and 3D-printed material for **a** breast lumpectomy, **b** soft-tissue sarcoma, and **c** liver tumor. The top image in each column is generated from the original DICOM rendered using 3D-Slicer. Each model contains a solid tumor which was printed with 1 μM ICG-equivalent material

Three anatomical shapes were printed based on exported DICOM data using custom resin formulations to mimic representative tissue optical properties (Fig. 2). The breast and sarcoma models consisted of three parts: two pieces of bulk material and a tumor inclusion, whereas the liver model had a surface tumor and only required two parts. A small rectangular block was 3D printed for each material using the same batch of resin and measured using SFDI to determine the optical properties provided in Table 2.

**Table 2:** Phantom optical property estimates at 800 nm

Optical properties	Breast tumor	Breast normal	Sarcoma	Liver
*μ*_*s*_′ (mm^−1^)	0.68	1.06	0.24	0.26
*μ*_*a*_ (mm^−1^)	0.013	0.005	0.006	0.143

**Fig. 3:**
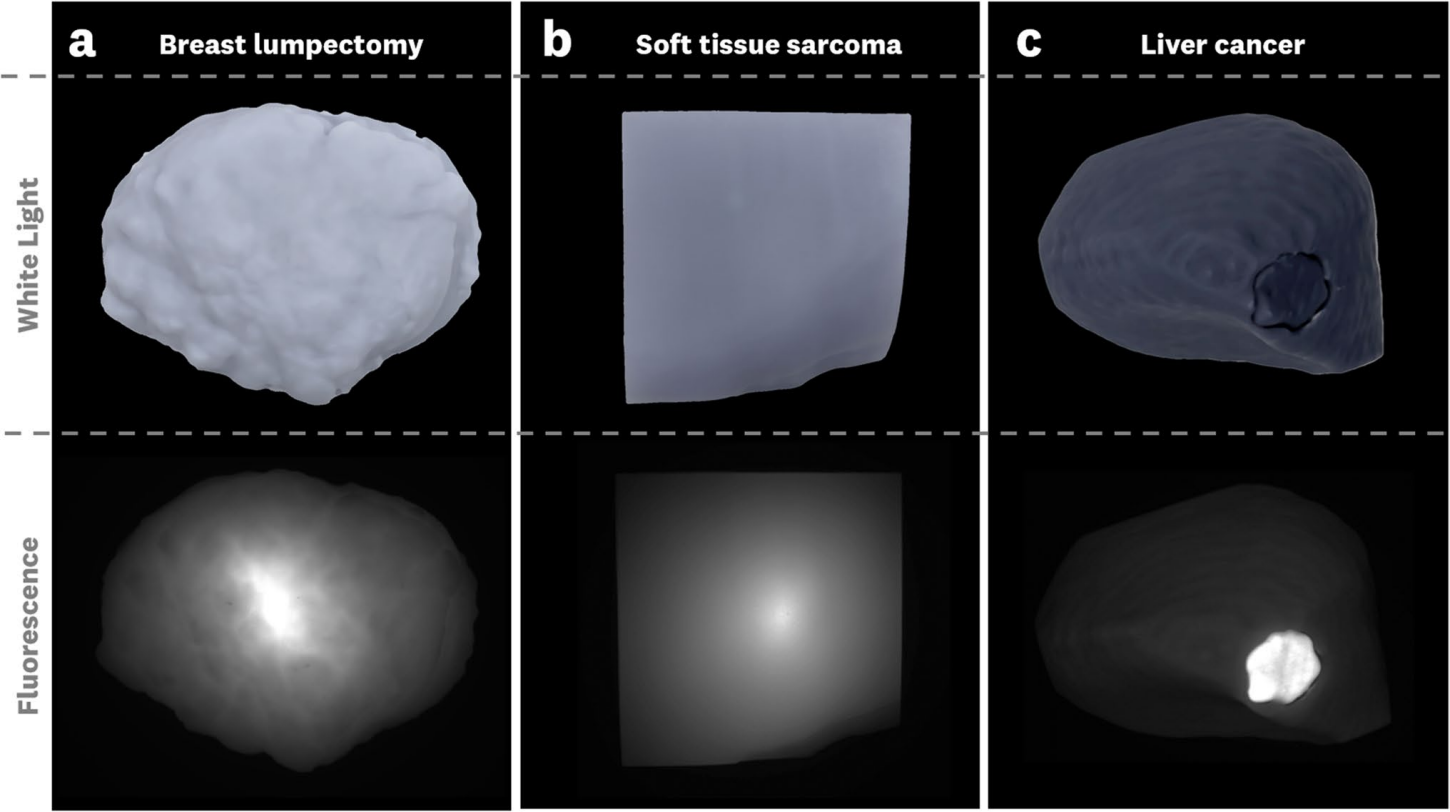
Representative top-down fluorescence images of the three tumor phantoms. Note that the window/level of intensity display was adjusted for each individual fluorescence image to facilitate visualization of the targets. Each phantom has distinct optical properties and contains 0.03 μM concentration ICG-equivalent fluorophore in the bulk material, and 1 μM in the tumor

Representative top-down fluorescence and white light images for the phantoms are shown in Fig. 3. The targets shown used the 3% background ICG to facilitate the visualization of the targets. From these images, we see variations in the fluorescence distributions that depend on both the surface geometries and the optical properties of each model.

Masks were used to collect statistics of the detected intensity in each region of interest. Since the compared systems have different bit depths, the analysis results are reported relative to the maximum intensity of the image. In the representative example of the breast lumpectomy (Fig. 1), the mean tumor intensity was 0.58 and 0.68 (SD: 0.15 and 0.18) for the FLUOBEAM and EleVision systems, respectively. Similarly, for the maximum intensity region, the mean intensity was 0.91 and 0.89 (SD: 0.06 and 0.13) for these systems. The background for both systems was equivalent, with a mean intensity of 0.05 (SD: 0.03) for the threshold-based region in each system, and a mean intensity of 0.04 (SD: 0.01) for both smaller background regions. Similar values were collected for each phantom and used to calculate the SBR, SNR, CNR and CVR. A range of these values for the two imaging systems, using both sets of masked regions, are provided in Table 3.

**Table 3:** Sensitivity metrics calculated for each phantom using both fluorescence imaging systems. *μ* = pixel mean. *σ* = pixel standard deviation. System 1 is the FLUOBEAM-800, and system 2 is the EleVision. No BG indicates the bulk material contained no fluorophore whereas 3% ICG BG contained 0.03 μM concentration ICG-equivalent fluorophore

			SBR $\frac{{\mu }_{T}}{{\mu }_{B}}$		SNR $\frac{{\mu }_{T}}{{\sigma }_{B}}$		CNR $\frac{{\mu }_{T}-{\mu }_{B}}{{\sigma }_{B}}$		CVR $\frac{{\mu }_{T}-{\mu }_{B}}{\sqrt{{\sigma }_{T}^{2}+{\sigma }_{B}^{2}}}$	
			Otsu ROI	Circle ROI	Otsu ROI	Circle ROI	Otsu ROI	Circle ROI	Otsu ROI	Circle ROI
Breast phantom	**No BG**	System 1	8.1	14	14	58	13	54	3.2	10
		System 2	8.3	11	15	37	13	34	3.3	5.8
	**3% ICG BG**	System 1	5.7	9.3	15	38	12	34	3.1	12
		System 2	6.1	9.9	15	59	13	53	2.8	9.3
Sarcoma phantom	**No BG**	System 1	5.7	11	14	21	11	19	3.6	16
		System 2	7.4	12	14	75	14	68	3.3	21
	**3% ICG BG**	System 1	4.9	7.1	14	50	11	43	3.6	27
		System 2	5.2	8.0	15	58	12	51	3.1	26
Liver phantom	**No BG**	System 1	5.6	5.7	12	27	9.7	30	5.5	3.0
		System 2	11	12	31	165	28	150	6.0	2.9
	**3% ICG BG**	System 1	7.4	7.8	14	52	12	45	6.1	2.8
		System 2	13	15	37	163	35	152	6.6	2.6

**Fig. 4:**
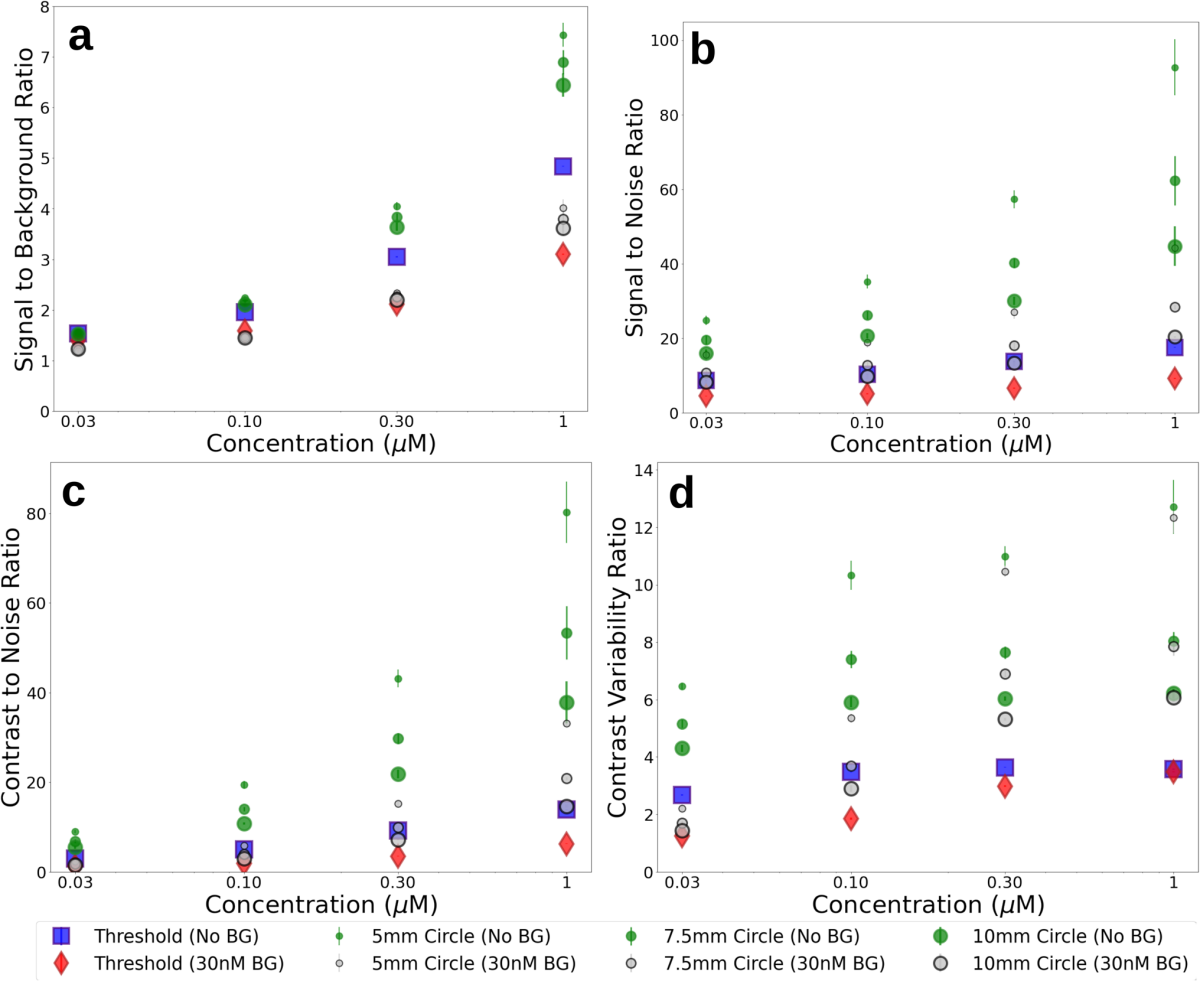
Image analysis metrics calculated from breast phantom fluorescence images using either Otsu threshold as described in the text, or a circular ROI of set diameter. Images were collected using bulk material containing either no fluorophore (No BG) or 0.03 μM concentration ICG-equivalent (30 nM BG). Metrics calculated are **a** SBR, **b** SNR, **c** CNR, and **d** CVR

To understand how the reported metrics vary with tumor concentration, additional measurements were collected of the breast model using a custom fluoresence imaging system. Both bulk materials with either no fluorophore or a 0.03-μM ICG-equivalent concentration were used, and tumor inclusions of 0.03 μM, 0.1 μM, 0.3 μM, and 1 μM were imaged in triplicate. Measurements were collected over 24 h by two different operators to demonstrate the repeatability of using a solid phantom model. The summary of the reported metrics is presented graphically in Fig. 4, where error bars represent the standard error between the calculated metrics over 3 observations.

## Discussion

Solid composite phantoms are increasingly being used for the performance characterization of fluorescence imaging systems. These designs generally use simple geometric shapes, which ease the ability to extract quantitative metrics. These reference targets and the resulting metrics are primarily used to quantify invariable system characteristics such as linearity, dynamic range, spatial resolution, and field homogeneity [14, 16, 26]. These factors are important for system comparisons and provide a useful reference to enhance reproducibility and enable multi-center studies. Yet, even with well-characterized systems, additional factors inhibit the ability to extract quantitative information from biological data.

Previous work with 3D-printed anthropomorphic optical phantoms has demonstrated a brain perfusion model using injected ICG as contrast [27]. Our current work extends this concept by using an ICG-equivalent fluorophore embedded in materials with a range of tissue simulating optical properties. While simple geometric phantoms provide ideal characterization metrics important to system engineering and design, anthropomorphic designs present an opportunity to study specific biological models and provide a mechanism to study image analysis techniques. As a training tool, the presented solid phantoms are not suitable for surgical manipulation, but anthropomorphic phantoms provide an opportunity for didactic surgical training related to interpreting fluorescence visualizations. While ICG is not indicated for solid tumor detection, targeted 800 nm channel probes are used for this function [8, 10, 28]. Clinical FGS primarily focuses on perfusion and vascularization assessment, which generally utilizes high ICG concentrations and does not require quantification. However, as new targeted contrast agents become available, there will be an increased need for acquiring quantitative data [29].

The commercial imaging systems used in this study provide representative examples of difficulties with extracting quantitative information from fluorescence images. The dynamic range of the FLUOBEAM provided a 16-bit monochrome TIFF image, whereas the EleVision uses dynamic auto-gain features to produce an 8-bit per channel color image using JPEG compression. The working distance for the two systems differed, but is an operational factor that should be considered. These operational variations are harder to track than the invariable system characteristics, but are a crucial component needed for advancing quantification efforts and enable multi-center reproducibility. The standard development working groups organized by professional societies (*e.g.*, AAPM TG311, ESMI, WMIS) should seek input on what operational parameters should be recorded during acquisition. Ideally, this information could be incorporated into metadata associated with acquired images to aid in the development of future quantification tools.

The biomimetic nature of the phantoms presented in this study demonstrate the uncertainty in extracting sensitivity metrics when image segmentation is not based on simple geometries. The metrics reported in Table 3 provide evidence on this uncertainty which can be biased based on the region of interest. The wide range of each metric was largely due to the different region definitions, where the 5-mm diameter region around the peak intensity skewed the reported metrics to much higher ratios. From the data provided in Fig. 4, it can be observed that simple intensity ratios, such as the SBR, have good agreement at lower tumor inclusion concentrations when comparing small ROIs with larger threshold regions. When background noise is considered, the small ROI may have insufficient statistical power, resulting in higher reported ratios.

The EleVision system provides a way to define points within the image to determine the intensity relative to the maximum fluorescence intensity. These values are inversely proportional to the SBR and extend the colormap visualization by providing a quantitative values. However, the system implements an auto-gain algorithm for visualization purposes, so the provided percentages are not comparable for different fields.

The CNR provides more context about detectability than the SNR because the CNR considers the intensity difference of the two regions. An example of where this is observed is with the breast lumpectomy and sarcoma models. Both models had similar SNRs when the bulk tissue had different fluorescence concentrations. This is likely due to the diffusion of fluorescence from the bulk material increasing the intensity of the tumor region, but offsetting the increase with higher background noise. The CNR of these models shows a decrease when background fluorescence is increased, indicating an expected drop in detectability.

The CVR goes one step further by considering the standard deviation, or noise, in each region. While the CVR plateaus with increasing tumor fluorescence, it represents discrimination between the two segments, and has been reported to be highly correlative with an “ideal observer” [25]. The observed plateau may be explained by the optical diffusion through a non-regular geometry, which is a challenge surgeons must also overcome when interpreting visualization. In phantoms and reference targets with simple geometric shapes, often used for used for system characterization, the importance of the CVR may be less apparent because there is minimal noise to consider, but these anthropomorphic phantom models highlight the utility of this metric.

Similar to the collection of image acquisition metadata, consensus is needed on which sensitivity metrics should be reported and best practices when defining regions of interest. Without a consensus, there is minimal value in reporting these metrics since they are so easily biased. As the field increasingly adopts fluoresence guided imaging to make clinical decisions, we need to make sure we can translate one study to another. In order to move the field forward, it is important to provide detailed methods on data acquisition and analysis method. Reporting a single metric of SBR, SNR, CNR, or CVR without reporting the value of variables used in the calculation introduces uncertainty when comparing study results and develop clinical best practices.

In additional to surgical guidance, preclinical fluorescence imaging systems are useful tools for studying pharmacokinetics in animal models, providing semi-quantitative data using a fixed working distance and a closed-boxed system to minimize interference from ambient light [30]. These imaging systems are also being introduced in some clinical studies for back-table imaging, which is especially useful in studying targeted fluorophores [8–10, 29]. However, more work is needed before quantitative imaging is a common tool in FGS, and additional training should be implemented to ensure images are not misinterpreted. Anthropomorphic phantoms with biomimicking optical properties are one tool to help demonstrate these complexities so further consensus can be built around the concept of quantitative fluorescence imaging. Future work should consider how additional fluorophores or specific anatomical designs could benefit from similar techniques. It will be important to consider the optical properties of these designs for the given fluoresence imaging wavelengths. Additionally, phantoms mimicking the mechanical properties of tissue would provide additional opportunity for surgical training.

## Conclusions

Optical phantoms are more than just a tool for system characterization. 3D printing techniques provide a platform for demonstrating complex biological models that introduce real-world complexities of quantifying fluorescence image data. Controlled iterative development of these phantom designs can be used as a tool to advance the field and provide context to consensus-building and educational initiatives.

## Supplementary Information

Below is the link to the electronic supplementary material.

Supplementary file1 (XLSX 9 KB)

Supplementary file2 (XLSX 16 KB)
